# *Thermus thermophilus* as source of thermozymes for biotechnological applications: homologous expression and biochemical characterization of an α-galactosidase

**DOI:** 10.1186/s12934-017-0638-4

**Published:** 2017-02-13

**Authors:** Martina Aulitto, Salvatore Fusco, Gabriella Fiorentino, Danila Limauro, Emilia Pedone, Simonetta Bartolucci, Patrizia Contursi

**Affiliations:** 1 Dipartimento di Biologia, Università degli Studi di Napoli Federico II, Complesso Universitario Monte S. Angelo, Via Cinthia, 80126 Naples, Italy; 2 Division of Industrial Biotechnology, Department of Biology and Biological Engineering, Chalmers University of Technology, Gothenburg, Sweden

**Keywords:** α-Galactosidase, *Thermus thermophilus*, Thermozymes, Recombinant expression, Themostability

## Abstract

**Background:**

The genus *Thermus*, which has been considered for a long time as a fruitful source of biotechnological relevant enzymes, has emerged more recently as suitable host to overproduce thermozymes. Among these, α-galactosidases are widely used in several industrial bioprocesses that require high working temperatures and for which thermostable variants offer considerable advantages over their thermolabile counterparts.

**Results:**

*Thermus thermophilus HB27* strain was used for the homologous expression of the TTP0072 gene encoding for an α-galactosidase (*Tt*GalA). Interestingly, a soluble and active histidine-tagged enzyme was produced in larger amounts (5 mg/L) in this thermophilic host than in *Escherichia coli* (0.5 mg/L). The purified recombinant enzyme showed an optimal activity at 90 °C and retained more than 40% of activity over a broad range of pH (from 5 to 8).

**Conclusions:**

*Tt*GalA is among the most thermoactive and thermostable α-galactosidases discovered so far, thus pointing to *T. thermophilus* as cell factory for the recombinant production of biocatalysts active at temperature values over 90 °C.

**Electronic supplementary material:**

The online version of this article (doi:10.1186/s12934-017-0638-4) contains supplementary material, which is available to authorized users.

## Background

After cellulose, the second most abundant biopolymer on earth is hemicellulose, an heterogeneous polymer of pentoses (xylose and arabinose) and hexoses (glucose, galactose, mannose) [1]. Among these, mannans comprise linear or branched polymers derived from sugars such as d-mannose, d-galactose and d-glucose. Moreover, they represent the major source of secondary cell wall found in conifers (softwood) and leguminosae [2]. Based on their sugar composition, mannans are classified in four subfamilies: i.e. mannans, glucomannans, galactomannans and galactoglucomannans. The concerted action of different hydrolytic enzymes such as β-glucosidases (EC 3.2.1.21), endo-mannanases (EC 3.2.1.78), mannosidases (EC 3.2.1.25) and α-galactosidases (EC 3.2.1.22) is needed to achieve the degradation of galactoglucomannans, the most complex mannans subfamily [3].

α-Galactosidases (α-d-galactoside galactohydrolase EC 3.2.1.22) are exoglycosidases that catalyse the cleavage of the terminal non-reducing α-1,6-linked galactose residues present in different galactose-containing oligo- and polysaccharides. α-Galactosidases are widely distributed in microorganisms, plants, animals and mammalians, including humans [4]. The localization of α-galactosidases can be cytoplasmic (e.g. *Escherichia coli)*, lysosomal (e.g. *Homo sapiens*) or extracellular (e.g. yeast) [5, 6].

α-Galactosidases have a great potential in both biotechnological and medical applications. For instance, these enzymes are used for the treatment of Fabry’s disease [7], in xenotransplantation [8] and in blood group transformation for safety transfusion [9]. Furthermore, α-galactosidases offer a promising solution for the degradation of raffinose in beet sugar industry [10], pulp and paper manufacturing [11] as well as in soy food [12] and animal feed processing [13].

The interest towards enzymes hydrolysing hemicellulose, such as α-galactosidases, has particularly increased in recent years. Indeed, they are extensively used in synergic combination with cellulases [14] for the production of bioethanol from lignocellulose [15]. In this regard, it is noteworthy that the pretreatment of raw lignocellulosic material requires elevated temperatures; therefore, thermostable and thermoactive enzymes are suitable for this purpose [16], since they can withstand high temperature, extreme pH and pressure, as well as the presence of some inhibitors, including toxic metals. Furthermore, as catalysts, thermostable enzymes might provide additional advantages, since higher temperatures often promote: (1) better enzyme penetration, (2) cell wall disorganisation of the raw materials, (3) increase of the substrate solubility and (4) reduction of contamination. Over all, these features may improve the global yield of the process [17].

In recent years, the thermophilic microorganism *T. thermophilus* has emerged has a rich source of polymer-degrading enzymes (e.g. α-amylases, xylanases, esterases, lipases, proteases and pullulanases). Indeed, this thermophilic bacterium is able to grow on different organic sources such as various proteinaceous and carbohydrates substrates [18].

Although *E. coli* has been successfully employed to produce thermophilic recombinant proteins/enzymes [19–24], in some cases their expression level can be too low to perform functional and structural characterization as well as to exploit their biotechnological potential [25–27]. For this reason, efficient and reliable “hot” expression systems are needed.

Some genetic systems for both archaeal and bacterial thermophilic microorganisms have been designed [28–32]. However, the amount of the recombinant proteins is in general lower than that achieved through conventional mesophilic expression systems [33].

A thermophilic expression system for *T. thermophilus* HB27 was previously developed and proved to be suitable to achieve high expression levels of heterologous proteins [34, 35]. Therefore, we have used this system to produce a novel α-galactosidase (named *Tt*GalA) from *T. thermophilus* HB27. In particular, the coding gene was over-expressed in the native host and the recombinant enzyme was purified and characterized. *Tt*GalA exhibits an optimal hydrolytic activity at high temperature (90 °C and pH 6.0), a good catalytic efficiency (k_cat_ = 709.7/s) and a significant thermostability (30 h at 70 °C), which are all interesting features for industrial applications.

## Results and discussion

The genus *Thermus* comprises thermophilic and hyperthermophilic bacteria [18], which represent genetic reservoirs of several thermozymes potentially useful for industrial bioprocesses at high temperature [36, 37]. This aspect, along with other intrinsic features such as the natural competence of many strains, high growth rates and biomass yields, make these thermophiles suitable models for the production of thermozymes [27, 38].

In this work we have used *T. thermophilus* HB27::*nar* as host for the homologous expression of a novel α-galactosidase (*Tt*GalA), one of the most thermoactive α-galactosidases identified so far.

### Sequence analysis

**Fig. 1:**
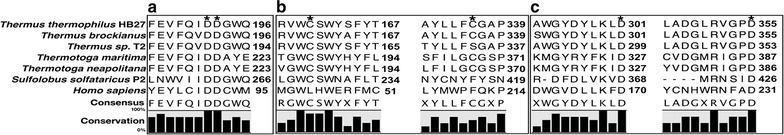
Sequence homology of α-galactosidases from different sources. The amino acid sequences of *Thermus thermophilus* HB27 (Accession No. AAS82402), *Thermus* sp. strain T2 (75%), *Thermus brockianus* (73%), *Thermotoga neapolitana* (36%), *Thermotoga maritima* (35%), *Sulfolobus solfataricus* (39%) and *Homo sapiens* (23%) were aligned for optimal sequence similarity using the program CLUSTAL W. **a** The consensus motif ([LIVMFY]-x(2)-[LIVMFY]-x-[LIVM]-D-D-x-[WY]) is characteristic of α-galactosidases; **b** amino acid stretches including two conserved cysteine residues; **c** amino acid stretches surrounding the aspartic acids responsible for the nucleophilic and acid–base catalytic mechanism. Conserved amino acids are highlighted with *asterisk* in *T. thermophilus* HB27 sequence

An in-depth survey of the carbohydrate-active enzymes database CAZy (http://www.cazy.org/) was carried out to identify putative α-galactosidases in the genome of *T. thermophilus* HB27. The only protein sequence retrieved is encoded by TTP0072 (Accession No. AAS82402) and shows high sequence identity with previously characterized α-galactosidases from *Thermus* sp. strain T2 (75%; Accession No. BAA76597), *Thermus brockianus* (73%; Accession No. AF135398), *Thermotoga neapolitana* (36%; Accession No. AF011400), *Thermotoga maritima* (35%; Accession No. AJ001776), *Sulfolobus solfataricus* (39%; Accession No. Q97U94) and *H. sapiens* (23%; Accession No. CAA29232.1). In general, sequence identities among the analysed proteins fall into regions that are “signature” of the catalytic activity and/or of the structural properties of α-galactosidases. A multiple alignment (Fig. 1a) revealed the presence of a consensus motif (FEVFQIDDGW) in the TTP0072 translated sequence characteristic of α-galactosidases. This is generally localised within the central region of bacterial enzymes (CAZy family GH36) or at the amino-terminal region of eukaryotic variants (CAZy family GH27). The presence of such a sequence points out to a common reaction mechanism and/or a similar substrate binding site [39]. Two aspartic residues i.e. D193–194 underlined in the above consensus sequence are followed by a cysteine or a glycine in the GH27 or GH36 members, respectively. Moreover, GH27 and GH36 families share another common feature, which is the presence of two additional aspartic acids involved in the acid–base catalytic mechanism (D301 and D355 in the protein from *T. thermophilus* HB27, Fig. 1). At consensus level, whereas the motif including the catalytic nucleophile (D301) is fully conserved [K(Y/V/L/W)**D**], the A/B aspartic acid motif (D355) is more variable (RXXX**D**) (Fig. 1c). The co-presence of these two motifs defines the sub-group identity of GH36bt (where “bt” stands for bacterial thermophilic) [40] that also includes the thermophilic *Thermotoga* and *Thermus* α-galactosidases. Another characteristic of the GH36bt enzymes is the presence of two conserved cysteine residues (C161 and C336) (Fig. 1b) whose functional, structural and stabilization role is questioned.

The TTP0072 gene is located on the megaplasmid pTT27 upstream of a gene encoding for the galactose-1-phosphate uridylyltransferase gene (*galT*) and partially overlapping it. This operon-like structure closely resembles that found in other *Thermus* spp. [41], thus suggesting that their functional association might be important for galactose metabolism.

### Cloning, expression and purification of *Tt*GalA

**Fig. 2:**
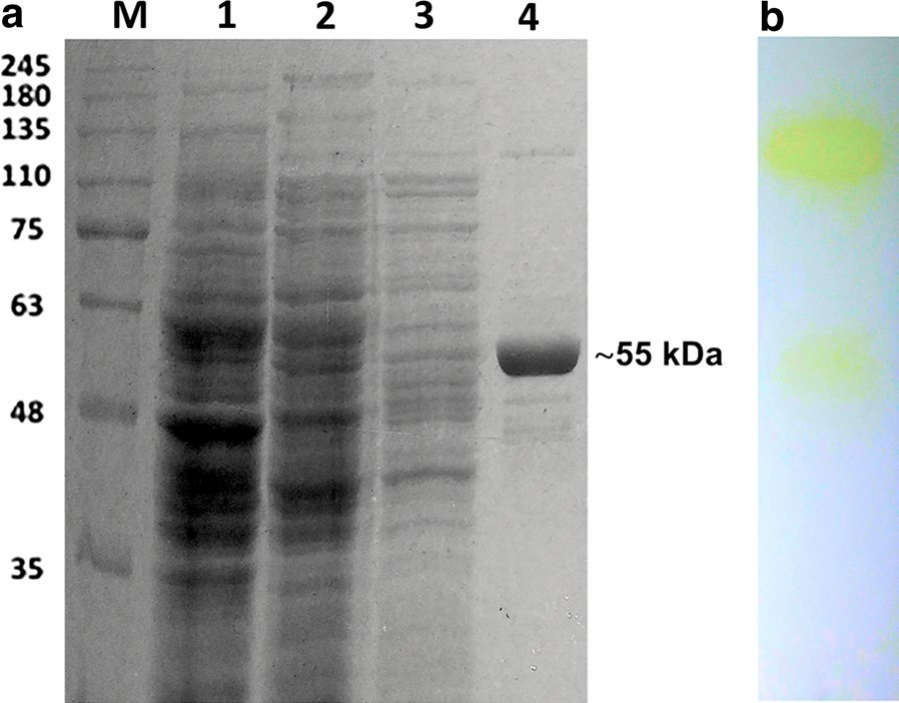
SDS-PAGE of the recombinant α-galactosidase *Tt*GalA fused to a His-tag at it N-terminal sequence. **a** M, molecular mass markers; 1, *T.thermophilus::nar* cellular extract not transformed; 2, *T.thermophilus::nar pMKE2*-*TtgalA* cellular extract after overnight growth; 3, anionic exchange chromatography; 4, His-Trap affinity chromatography; **b** zimography

The TTP0072 gene was cloned both in pET28(b) and in pMKE2 vectors to compare the expression levels of the N-terminal His-tagged enzymes *Ec*GalA and *Tt*GalA in the heterologous (*E. coli*) as well as in the homologous (*T. thermophilus* HB27::*nar)* hosts. In the case of *E. coli*, after a two-step purification procedure i.e. a thermal precipitation at 70 °C and an affinity chromatography through His-trap column, the final protein amount was very low (0.5 mg/L) (Fig. 2a) (Additional file 1: Table S1).

On the other hand, the enzyme *Tt*GalA was expressed at higher level in *T. thermophilus* HB27::*nar* with a final amount of 5 mg/L (tenfold higher than *Ec*GalA). Moreover, its specific activity (338 U/mg) was twofold higher than that of *Ec*GalA (Additional file 1: Tables S1, S2). As previously observed, the over-production of soluble and active enzymes in mesophilic hosts is in some cases limited by: (1) differences in the codon usage [42]; (2) domain misfolding during protein synthesis at a temperature (37 °C), which is far lower than the optimal growth temperature of the native host; (3) requirement of specific chaperone(s), cofactors, metals as well as genus- or species-specific post-translational modifications [27].

Given the significant higher amount of the soluble active enzyme produced by the thermophilic host, all the subsequent characterizations were performed on the *Tt*GalA enzyme. The *nar* promoter, which drives the expression of the genes cloned in the pMKE2 vector, is induced by the combined action of nitrate and anoxia in the facultative anaerobic derivatives of *T. thermophilus* HB27 [34]. In our tested conditions, the biomass yield of the culture achieved under anaerobic growth was rather low, thus negatively affecting the amount of enzyme produced by the cells. To overcome this limitation, the expression of *Tt*GalA was carried out by growing *T. thermophilus* HB27::*nar* cells aerobically to reach an higher biomass yields. A total protein amount, similar to that of the cells cultured anaerobically, was achieved.

We resolved to set up a purification protocol from crude extracts of aerobically cultured cells based on anionic exchange followed by affinity His-trap chromatography. The recombinant enzyme purified to homogeneity displays a single band on SDS-PAGE with an estimated molecular weight (MW) of 55 kDa (Fig. 2a), which is consistent with the predicted MW of a his-tagged monomer (55.8 kDa). The identity of the recombinant *Tt*GalA was verified by mass spectrometry (data not shown). It is important to note that the presence of the His-tag at the N-terminus of the recombinant enzyme ensures that the reported experiments are not affected by the presence of the endogenous enzyme. Furthermore, zymography revealed the presence of two bands with hydrolytic activity indicating that: (1) *Tt*GalA adopts an oligomeric structure; (2) the oligomer is far more active than the monomer and (3) it is partially resistant to the denaturing conditions employed in the SDS-PAGE. These results prompted us to further investigate on the quaternary structure of *Tt*GalA (Fig. 2b).

### Characteristics of the recombinant *Tt*GalA

#### Determination of the molecular weight

To assess the quaternary structure, size-exclusion chromatography coupled with a triple-angle light scattering-QELS, was performed. This analysis showed a molecular weight of about 320 kDa ± 0.2% (RH = 8.1 nm ± 3%), thus indicating that *Tt*GalA is a hexamer in solution. This oligomeric structure is in agreement with that of some previously characterised α-galactosidases, which adopt dimeric (*Thermotoga maritima*) [43], trimeric (*Sulfolobus solfataricus*) [40], tetrameric (*T. brockianus*) [39], octameric (*Thermus* sp. *strain T2*) structure [44]. This complex oligomeric state might correlate with the stability at higher temperature of *Tt*GalA, as it was showed for *B. stearothermophilus* α-galactosidases [45].

To understand if C161 and C336 had a structural role (Fig. 1b), the purified *Tt*GalA was analysed on SDS-PAGE with and without a reducing agent. In the sample containing β-mercaptoethanol *Tt*GalA is present mainly in monomeric form, while in the absence of this reducing agent the protein forms essentially high MW oligomers (data not shown). Indeed, the widespread stabilizing role of intracellular disulphide bonds in thermophiles and hyperthermophiles, has been already established as a strategy for protein stabilization [46].

#### Catalytic and stability properties

*Tt*GalA is able to hydrolyze *p*NP-α-d-galactopyranoside, but shows negligible activity on both *p*NP-α-substituted (d-glucose, d-mannose, l-rhamnose) and *p*NP-β-substituted (d-galactose, d-glucose, and d-mannose) (Table 1). In particular, the enzyme has a barely detectable activity for β anomer of galactose (6.75 U/mg), which is 50-fold lower than activity over *p*NP-α-galactose (338.0 U/mg). Therefore, the kinetic enzymatic properties were determined, using *p*NP-α-d-galactopyranoside as substrate, at optimal pH and temperature (Table 2). *Tt*GalA shows higher affinity towards its substrate (K_M_ = 0.69 mM) compared to α-galactosidase from *Thermus* sp. strain T2 (K_M_ = 4.7 mM) [44] and *T. brockianus* (K_M_ = 2.5 mM) [39]. However, comparing the kinetic parameters between the most known thermoactive (T_opt_ 105 °C) α-galactosidase of *Thermotoga neapolitana* (*Tn*GalA) and the *Tt*GalA, the K_M_ value is very similar. Interestingly, the different catalytic constant of 152.5/s towards 709.7/s, for *Tn*GalA and *Tt*GalA respectively [47] (Table 2) reflects a great efficiency of *Tt*GalA on *p*NP-α-d-galactopyranoside substrate. This aspect constitutes an important criterion for employing different enzyme variants for industrial purposes. For istance, the high catalytic efficiency of *Tt*GalA makes it a suitable candidate to enhance pulp bleachability in combination with other hemicellulases exhibiting different catalytic specificity [11].

**Table 1:** Substate specificity of *Tt*GalA

Substrate	Specific activity (U/mg)
*p*NP-α-d-mannose	0
*p*NP-β-glucose	0.65
*p*NP-α-l-rhamnose	1.5
*p*NP-α-d-glucose	2.16
*p*NP-β-galactose	6.75
*p*NP-β-mannose	7.13
*p*NP-α-d-galactose	338.0

**Table 2:** Kinetic parameters for the hydrolysis *p*NPG hydrolysis at 90 °C by the *Tt*GalA

K_M_ (mM)	V_max_ (U/mg)	k_cat_ (/s)	k_cat_/K_M_ (/s M)
0.69 ± 0.017	338.0 ± 7.9	709.7 ± 17.7	1.03 × 10^4^ ± 0.025 × 10^4^

**Fig. 3:**
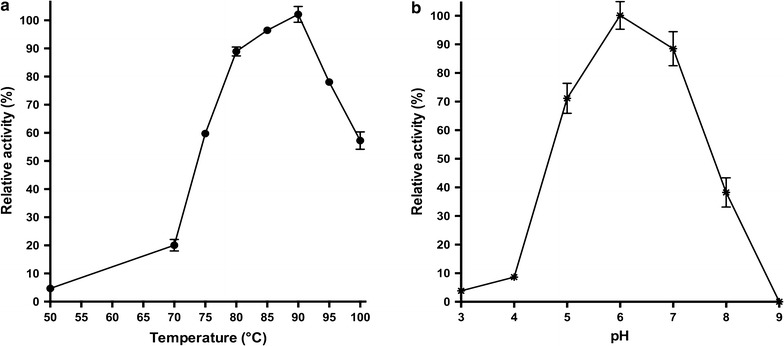
**a** Determination of the *Tt*GalA catalytic activity in the temperature range of 50 to 100 °C. *p*NP-α-d-galactopyranoside was used as substrate dissolved in 50 mM sodium phosphate buffer pH 6.0. Relative activity at 90 °C was considered as 100%. **b** The relative activity was measured between pH 3.0 and pH 9.0, at 90 °C considering as 100% the activity at pH 6.0

**Fig. 4:**
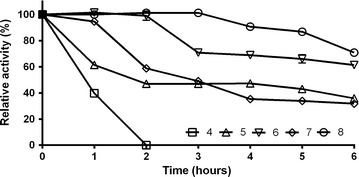
pH stability of *Tt*GalA. The enzyme was incubated in various buffers (pH 4–8) and aliquots of different time intervals were used for the residual activity assay

*Tt*GalA is among the most thermoactive and pH tolerant α-galactosidases known so far. Indeed, when assayed at different pH and temperatures, it exhibited an optimal hydrolytic activity at 90 °C and pH 6.0 (Figs. 3, 4). As reported for an α-galactosidase from *Bacillus megaterium* VHM1 (optimal pH 7–7.5) [48], it is possible to foresee the employment of *Tt*GalA for the removal of oligosaccharides from soya based foods, thus improving their nutritive value. Noteworthy, *Tt*GalA might be a better catalyst for this process, since its pH optimum (pH 6.0) is even closer to that of the soymilk hydrolysis process (pH 6.2–6.4).

Interestingly, the retained activity was greater than 40% within the pH range from 5.0 to 8.0, which is a quite wide tolerance range compared to other characterised thermostable α-galactosidases [40]. Therefore, pH shifts during on-going enzymatic reaction in industrial processes could have a minor impact on its activity (Fig. 3). The recombinant enzyme did not lose activity after 2 h of incubation at pH 6.0 and 8.0, and it retained up to 60% of residual activity after 6 h (Fig. 4). Approximately 50–60% loss in activity was recorded on either side of the pH optimum after 6 h incubation, while at pH 4.0, the activity rapidly dropped, as expected from the pH-dependence data (Fig. 4). Because of its considerable tolerance towards neutral/slightly alkaline pH values, *Tt*GalA could be employed in hydrothermal processing in which water in liquid phase or in vapour phase is used to pretreat lignocellulosic biomasses [49]. In this process, controlling the pH around neutral values minimizes the formation of fermentation inhibitors [50]. Typically, the optimal temperature for catalysis of thermophilic enzymes mirrors the growing temperature of the native host, like the α-galactosidase from *Thermus* sp. strain T2 (75 °C) [44], whereas their activity is limited at lower temperatures. *Tt*GalA is unusual since its optimum was set at 90 °C and its activity was lower at temperature ≤70 °C, which is the optimal growth temperature for most of *Thermus* species. Moreover, *Tt*GalA is among the most thermophilic α-galactosidases so far characterised, such as that of *T. neapolitana* (105 °C) [47], *T. brockianus* (94 °C) and *T. maritima* (95 °C) [39, 43].

**Fig. 5:**
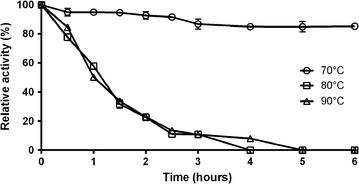
Thermal inactivation of *Tt*GalA. The purified enzyme from *T. thermophilus* was incubated in 20 mM sodium-phosphate buffer pH 6.0 at 90, 80 and 70 °C for different period of time and then assayed for residual activity at 90 °C

The thermal inactivation data indicate that the *Tt*GalA has a half-life of 60 min (Fig. 5) at its optimal temperature (90 °C). Moreover, residual activity higher than 90% was detected up to 6 h of incubation at 70 °C (Fig. 5), retaining 50% of its activity after 30 h (not shown).

It has already been reported that previously characterised enzymes from *Thermus* species display in vitro an optimal catalytic temperature higher than the growing temperature of the native host [51], thus making this microorganism a fruitful source of enzyme catalytically active at temperature above 70 °C. Due to the elevated temperatures used during some industrial applications (such as sugar manufacturing processes and/or raw material pretreatments in bioethanol production), stability at high temperatures is an important feature for the utilization of α-galactosidases, since it prevents the loss of ternary/quaternary structures that leads to enzyme inactivation as for the mesophilic counterparts [36, 37].

#### Effect of metal ions

Metal ions can be released during processing of biomass as consequence of corrosion of pretreatment equipment, resulting in the liberation of heavy metal ions, which can be inhibitory to biocatalysts [52]. Moreover, other cations can derive from chemicals used to adjustment the pH. Noteworthy, several divalent cations are potent inhibitors of α-galactosidases; therefore, we tested their effect on the enzyme activity over a range of concentration from 0.5 to 5 mM (Table 3). Interestingly, the enzyme turned to be slightly activated in the presence of Co^+2^, Mn^+2^, Zn^+2^ at 1 mM concentration suggesting that it is a metalloenzyme or requires divalent cations as cofactors. On the other hand, this effect is negligible with monovalent cations (Table 3). The inhibition effect occurred only at the highest concentration of metal ions tested (5 mM). To further investigate on the possible role of metal ions, the enzyme activity was assayed in the presence of EDTA (5 mM) and a 20% reduction of its catalytic activity was observed. The inhibitory effect of EDTA was studied up to 40 mM concentration (not shown) resulting in a linear decrease of the enzymatic activity, thus indirectly confirming the role as cofactor of the metal ions (Co^+2^, Mn^+2^, Zn^+2^) (Table 3). Similar to these findings, EDTA slightly inhibited also the α-galactosidase activity from *Lenzites elegans* [53], whereas it has no effect on the activity of α-galactosidases from *B. megaterium* and *Ganoderma lucidum* [48, 54].

**Table 3:** Influence of metal ions on the relative activity of *Tt*GalA

Metal ions	0.5 mM	1 mM	2.5 mM	5 mM
Mg^+2^	99.3 ± 0.7	91.7 ± 0.3	89.9 ± 2.5	55.6 ± 0.5
Ca^+2^	95.9 ± 0.7	95.8 ± 0.1	90.1 ± 2.4	27.8 ± 0.4
Cu^+2^	87. 7 ± 3.1	77.2 ± 1.6	63.9 ± 0. 97	22.2 ± 0.3
Li^+^	106.3 ± 0.5	113.9 ± 3.6	114.8 ± 3.1	93.6 ± 2.5
Zn^+2^	100.6 ± 2.2	110.3 ± 0.6	70.4 ± 0.6	15.1 ± 0.3
Mn^+2^	103.4 ± 1.0	128.5 ± 1.9	114.8 ± 3.0	20.2 ± 0.6
Co^+2^	101.8 ± 2.6	136.2 ± 0.4	92.8 ± 0.9	30.1 ± 0.7

#### Inhibition of activity

Since detergents are reported to strongly inhibit the α-galactosidase activity in *Glycine max* and *Pencillium griseoroseum* [55, 56], we assayed *Tt*GalA in the presence of 5 mM detergents. The enzyme turned out to be very sensitive to the common anionic detergent SDS, which leads to a complete loss of function possibly due to the disruption of enzyme native structure. By contrast, the non-ionic detergents Tween 20 and Triton X-100 had a less marked effect with reduction of the enzyme activity to ~40 and ~19%, respectively (Table 4).

**Table 4:** Influence of additives on the activity of *Tt*GalA

Compound (5 mM)	Relative activity (%)
EDTA	79.9
d-Galactose	27.9
d-Saccarose	44.6
d-Arabinose	54.7
Urea	60.0
Guanidine chloride	36.8
SDS	1.6
Tween 20	39.8
Triton X100	18.8

During pretreatment and saccharification of lignocellulosic biomasses, several sugars are released. These can inhibit the activity of glycoside hydrolases during the saccharification phase [49, 50]. Moreover, various sugars were also reported to inhibit the α-galactosidase activity, for instance, an α-galactosidase from *Aspergillus nidulans* is competitively inhibited by d-galactose and d-glucose [57]. Accordingly, the activity of *Tt*GalA turned out to be inhibited by the presence of several saccharides, such as d-galactose, d-saccarose and d-arabinose (Table 4). Nevertheless, TtGalA might have a potential use in the sugar beet industry for raffinose hydrolysis, because it retains 44.6% of its activity in presence of sucrose [10].

Finally, *Tt*GalA was assayed in presence of caotropic agents such as urea and guanidine chloride. The partial reduction of the catalytic activity is in agreement with its intrinsic stability as thermozyme [20].

## Conclusions

In this work, we report the biochemical characterization of a thermoactive and thermostable α-galactosidase from *T. thermophilus HB27* (*Tt*GalA). Moreover, the drawbacks of using a heterologous mesophilic host (*E. coli*) for the production of this thermozyme have been highlighted. Indeed, “hot” expression systems are in some cases indispensable to get functional thermozymes in reasonable amounts.

Interestingly, the long-term retained activity of *Tt*GalA (30 h at 70 °C) might pave the way to its utilization after thermal pretreatment of lignocellulosic biomass (pre-saccharification), when the temperature is still too high for the fungal enzymes currently used for the hydrolysis of the biomass.

Despite its already interesting catalytic features, a fine-tuning of *Tt*GalA enzymatic properties, through genetic engineering, will be attempted to make it even more suitable for industrial applications.

## Methods

### Bacterial strains and growth conditions

*Thermus thermophilus HB27* strain was purchased from DSMZ. A frozen (−80 °C) stock culture was streaked on a *Thermus* Medium (TM) solidified by the addition of 0.8% Gelrite® (Sigma) and incubated at 70 °C overnight [58]. *T. thermophilus* HB27::*nar* strain, kindly provided by Prof. J. Berenguer (Universidad Autónoma de Madrid) was used for the homologous expression of *Tt*GalA. *E. coli* strains were grown in Luria–Bertani (LB) medium at 37 °C with 50 μg/ml kanamycin, 33 μg/ml chloramphenicol as required. *E. coli* DH5α and BL21-CodonPlus (DE3)-RIL (Stratagene, La Jolla, CA, USA) strains were used for DNA manipulations and for the heterologous expression of the recombinant α-galactosidase, respectively.

### Cloning and sequencing of TTP0072 gene

A single colony of *T. thermophilus* HB27 was inoculated into TM liquid medium and genomic DNA was isolated using DNeasy® 124 Tissue kit, (Qiagen), according to the instruction manufacturer. TTP0072 gene, encoding for a putative α-galactosidase, was amplified by PCR from *T. thermophilus* HB27 genomic DNA using the primers 5′GGAGGGCATATGAGGCTGAA3′ (*Nde*I site underlined) and 5′CGGTGGAAGCTTTTATAGAAGG3′ (*Hin*dIII site underlined) and Phusion Taq Polymerase (NEB). The amplification was carried out at 94 °C for 1 min, 58 °C for 1 min and 72 °C for 1 min, for 35 cycles. The PCR product was purified with QIAquick PCR purification kit (Qiagen Spa, Milan, Italy) and cloned in pCR4-TOPO_vector using the TOPO TA CLONING Kit (Invitrogen). The identity of the cloned DNA fragment was confirmed by DNA sequencing (BMR Genomics). Then, the insert was sub-cloned into the *Nde*I/*Hin*dIII digested pET28(b) and for pMKE2 [34] vectors for *E. coli* and *T. thermophilus* HB27::*nar* expression, respectively.

### Expression and purification of recombinant *Ec*GalA and *Tt*GalA

The recombinant α-galactosidases expressed in *E. coli* (*Ec*GalA) and *T. thermophilus* HB27::*nar* (*Tt*GalA) bear an His-tag at their N-terminus. To express *Ec*GalA, *E. coli* BL21-CodonPlus (DE3)-RIL was transformed with the recombinant plasmid pET28(b)-*EcgalA*. Protein expression was induced by adding 0.5 mM of isopropil-β-d-1-thiogalactopyranoside (IPTG) to exponentially growing cells (0.5 OD_600_) and culturing them for 12 h. Since *Ec*GalA was poorly expressed, different approaches were attempted to achieve a sufficient amount of soluble recombinant protein: (1) varying the induction time (2, 4, 6, and 8 h and overnight induction); (2) lowering the temperature during induction (down to 20 °C); (3) decreasing the IPTG concentration (0.01–0.1 mM). At every conditions, the expression levels were monitored by SDS-PAGE and enzymatic assays. However, none of these strategies turned out to reasonably increase the final amount of the recombinant protein.

For the homologous expression 200 ng of pMKE2-*TtgalA* plasmid were added to exponentially growing (0.4 OD_600nm_) *T. thermophilus* HB27::*nar* cells and transformants were selected on TM plates at 70 °C containing 50 μg/ml kanamycin [59]. The induction of *Tt*GalA expression was performed as previously described [34].

Crude extracts from both *E. coli* BL21-CodonPlus (DE3)-RIL and *T. thermophilus* HB27::*nar* cells were prepared following a similar procedure. Cell pellets were collected from 1-L cultures by centrifugation at 4000×*g* for 15 min at 4 °C and resuspended in buffer A (50 mM Tris–HCl, pH 7.5 and 500 mM NaCl) for *Ec*GalA and in buffer B (50 mM Tris–HCl, pH 7.5) for *Tt*GalA purification, respectively. Protease inhibitor cocktail tablets (Roche) were added in both cases. The cells were homogenized by sonication (Sonicator:Heat System Ultrasonic, Inc.) for 5 min, alternating 30 s of pulse-on and 30 s of pulse-off and clarification of the cell extract was obtained by centrifugation at 40,000×*g* for 20 min at 4 °C. Purification of *Ec*GalA from the soluble fraction was performed through a two-step procedure, i.e. (1) thermal precipitation at 70 °C for 10 min followed by centrifugation at 5000×*g* for 20 min at 4 °C; (2) affinity chromatography on a His-Trap column (1 ml, GE Healthcare) connected to an AKTA Explorer system (GE Healthcare). The affinity chromatography was equilibrated with buffer A and the *Ec*GalA was eluted with the same buffer A supplemented with a linear gradient of imidazole (0–250 mM).

*Tt*GalA was purified through a similar procedure, except that the first thermal precipitation step was substituted with an anionic exchange chromatography on a Hi-trap Q HP column (5 ml, GE Healthcare). The column was equilibrated in buffer B and elution was performed in the same buffer through a linear gradient from 0 to 500 mM NaCl. The affinity chromatography was carried out under the same conditions described above. Protein identity was further verified by Western blot analysis using anti-His antibodies and LC–MS/MS analysis.

Protein concentration was estimated by using bovine serum albumin as standard according to Bradford [60]. Protein fractions displaying α-galactosidase hydrolysing activity toward *p*-nitrophenyl-α-d-galactopyranoside (herein named *p*NP-α-d-galactopyranoside, Sigma) were pooled, dialyzed against 20 mM Tris–HCl pH 7.5 and analysed by 12% SDS-PAGE [61]. The *Tt*GalA activity was detected through zimography in 12% SDS-PAGE under not reducing conditions. After electrophoresis, the gel was soaked in 2.5% Triton X-100 for 30 min at 4 °C and then was incubated with 20 mM of *p*NP-α-d-galactopyranoside solution at 90 °C for 10 min.

### Molecular weight determination

The native molecular weight of the purified *Tt*GalA was analysed by gel-filtration chromatography connected to Mini DAWN Treos light-scattering system (Wyatt Technology) equipped with a QELS (quasi-elastic light scattering) module mass value and hydrodynamic radius (*R*h) measurements [62]. Five hundreds micrograms of protein (1 mg/ml) were loaded on a S200 column (16/60, GE Healthcare) with a flow-rate of 0.5 ml/min and equilibrated in buffer A. Data were analysed using Astra 5.3.4.14 software (Wyatt Technology).

### Determination of the α-galactosidase activity

The *Tt*GalA standard assay was performed by using *p*NP-α-d-galactopyranoside as substrate (2.0 mM) in 50 mM sodium phosphate buffer (pH 6.0) at 90 °C in 160 μl of the reaction mixture using 50 ng of the enzyme. All assays were performed in triplicate in a 96-well microplate reader (Synergy H4, Biotek), on at least 3 different enzyme preparations. The reaction was stopped, after 10 min, by addition of 1 volume of cold 0.5 M Na_2_CO_3_ and the concentration of the released *p*-nitrophenol (molar extinction coefficient, 18.5/mM cm) was determined by measuring A_405nm_. One unit of enzyme activity (U) was defined as the amount of enzyme required to release 1 μmol *p*-nitrophenol per minute, under the above assay conditions.

#### Catalytic and stability properties

The optimal pH and temperature were determined by performing the *p*NP-α-d-galactopyranoside assay between pH 3.0–9.0 using the following buffers (each 50 mM): citrate phosphate (pH 3.0–5.0), sodium phosphate (pH 6.0–9.0). Thermal inactivation assays were performed by incubating 50 ng of enzyme at different temperatures (70, 80, 90 °C) in buffer sodium phosphate at pH 6.0 and taking aliquots at regular time intervals to measure the residual enzyme activity under standard assay conditions (90 °C for 10 min, pH 6.0). To test enzyme stability to pH, the assays were performed by incubating *Tt*GalA at 70 °C in various buffers (pH 4.0–8.0). The residual activity was measured at different time intervals following the α-galactosidase standard assay.

#### Inhibition of activity

The effect of MgCl_2_, CaCl_2_, CuCl_2_, LiCl, ZnSO_4_, MnCl_2_, CoSO_4_ on the α-galactosidase activity was studied over a range of concentrations (0.5–5.0 mM) by pre-incubating the enzyme (50 ng) for 5 min at room temperature with the specific metal ions and by measuring the residual activity under standard assay conditions.

The influence of reducing (DTT, β-mercaptoethanol) and chelating (EDTA) agents as well as of sugars (d-galactose, sucrose, l-arabinose, and d-fucose), each tested at 5 mM concentration, was studied under the same pre-incubation and assay conditions as above.

#### Substrate specificity

*Tt*GalA was tested for the hydrolysis of *p*NP-α-substituted hexoses (d-glucose, d-mannose, l-rhamnose) and of *p*NP-β-substituted hexoses (d-galactose, d-glucose, and d-mannose) at a concentration of 2 mM under standard conditions (90 °C and pH 6.0 for 10 min). The kinetic parameters were determined using different *p*NP-α-d-galactopyranoside concentration (ranging from 0.0125 to 2 mM) with 50 ng of *Tt*GalA for 3 min. The Michaelis constant (K_M_) and V_max_ were calculated by non-linear regression analysis using GraphPad 6.0 Prism software.

## Additional file

**Additional file 1: Table S1.** Purification table of *Ec*GalA. **Table S2.** Purification table of *Tt*GalA.

## Abbreviations

bt: bacterial thermophilic
CAZy: carbohydrate-active enzymes
DTT: dithiothreitol
*Ec*GalA: *Thermus thermophilus* α-galactosidase expressed in *E. coli*
EDTA: ethylenediaminetetraacetic acid
GH: glycoside hydrolase
*g*: gravity
h: hour(s)
IPTG: isopropil-β-d-1-tiogalattopiranoside
k_cat_: catalytic constant
K_M_: michaelis constant
LB: Luria-Bertani broth
LC–MS/MS: liquid chromatography-tandem mass spectrometry
*p*NP: P-nitrophenol
QELS: quasi-elastic light scattering
*R*h: hydrodynamic radius
s: second(s)
TM: thermus medium
*Tn*GalA: *Thermotoga neapolitana* α-galactosidase
*Tt*GalA: *Thermus thermophilus* α-galactosidase
SDS-PAGE: sodium dodecyl sulphate-polyacrylamide gel electrophoresis
