# Supplementary material for: Thermus thermophilus as source of thermozymes for biotechnological applications: homologous expression and biochemical characterization of an α-galactosidase
Source: Microb Cell Fact. 2017 Feb 13;16:28. doi: 10.1186/s12934-017-0638-4 (PMC5307791; doi:10.1186/s12934-017-0638-4)
Supplement: Supplementary file 1 — Additional file 1: Table S1. Purification table of EcGalA. Table S2. Purification table of TtGalA. [file 12934_2017_638_MOESM1_ESM.docx]

| ***Ec*GalA Purification step** | **Total protein  (mg)** | **Total activity  (U)** | **Specific activity**  **(U · mg-1)** | **Yield  (%)** |
| --- | --- | --- | --- | --- |
| **Cellular extract** | 100.5 | 1256.2 | 12.5 | 100.0 |
| **Thermal precipitation** | 10.7 | 502.3 | 46.9 | 40.0 |
| **Affinity chromatography** | 0.5 | 79.6 | 159.2 | 6.3 |

**Table S1.** Purification table of *Ec*GalA.

| ***Tt*GalA**  **Purification step** | **Total protein  (mg)** | **Total activity  (U)** | **Specific activity**  **(U · mg-1)** | **Yield  (%)** |
| --- | --- | --- | --- | --- |
| **Cellular extract** | 82.5 | 9004.8 | 109.1 | 100.0 |
| **Anionic exchange chromatography** | 35.7 | 8603.7 | 241.0 | 95.5 |
| **Affinity chromatography** | 5.0 | 1690.0 | 338.0 | 18.8 |

**Table S2.** Purification table of *Tt*GalA.
